# Biologging and biotelemetry tools bridge behaviour and physiology to advance the conservation and management of wildlife

**DOI:** 10.1093/conphys/coag061

**Published:** 2026-08-31

**Authors:** Steven J Cooke, Maria Thaker, Jessica Kendall-Bar, Emily S Choy, Emily Bennitt, Kim Birnie-Gauvin, Morgan L Piczak, L Monica Trondrud, Luc LaRochelle, Shane K Maloney, Andrea Fuller

**Affiliations:** Department of Biology and Institute of Environmental and Interdisciplinary Science, Carleton University, 1125 Colonel By Dr., Ottawa, ON K1S 5B6, Canada; Centre for Ecological Sciences, Indian Institute of Science, Bengaluru 560012, India; Center for Marine Biotechnology and Biomedicine, Scripps Institution of Oceanography, University of California San Diego, 9500 Gilman Drive, La Jolla, CA, USA; Department of Biology, McMaster University, 1280 Main Street West, Hamilton, ON L8S 4K1, Canada; Okavango Research Institute, University of Botswana, Shorobe Road, Maun, Botswana; Section for Freshwater Fisheries and Ecology, Technical University of Denmark, Vejlsøvej 39, Silkeborg 8600, Denmark; Department of Forest and Conservation Science, University of British Columbia, 2424 Mail Mall, Vancouver, BC V6T 1Z4, Canada; Department of Wildlife Ecology and Behaviour, National Research Institute for Agriculture, Food and Environment Occitanie-Toulouse, 24, Chemin de Borde Rouge, Auzeville-Tolosane 31320, France; Department of Biology and Institute of Environmental and Interdisciplinary Science, Carleton University, 1125 Colonel By Dr., Ottawa, ON K1S 5B6, Canada; School of Human Sciences, The University of Western Australia, 35 Stirling Highway, Crawley, WA, Australia; Brain Function Research Group, Department of Physiology, Faculty of Health Sciences, University of the Witwatersrand, 7 York Road, Johannesburg 2193, South Africa; Brain Function Research Group, Department of Physiology, Faculty of Health Sciences, University of the Witwatersrand, 7 York Road, Johannesburg 2193, South Africa

**Keywords:** Animal biology, biologging, biotelemetry, conservation, sensors, threats

## Abstract

The early days of animal tagging and tracking using electronic tags were largely restricted to biologists who were electronics hobbyists capable of creating their own devices. Studies were typically characterized by low sample sizes due to lack of storage, large tags with short lifespans and fickle technology, yet those early studies were also important in revealing what was possible. Today, there is an ever-expanding array of commercially available biologging and biotelemetry tools that employ a wide range of sensors that enable us to study the behaviour and physiology of animals, from insects to whales, from the depths of the oceans to high in the atmosphere. The use of multiple sensors can allow us to explore animal–environment interactions and better inform conservation and management. From the identification of problems to the testing of solutions, biologging and biotelemetry are ideal for generating robust, ecologically relevant and management-relevant data. In this perspective article, we first briefly summarize the history of biologging and biotelemetry and consider the ethical aspects of the use of these tools. We then use the Conservation Measures Partnership IUCN Threat Classification and Conservation Action Classification to summarize the key ways in which these tools can be applied to support conservation and management. Finally, we identify opportunities to further advance the ability of those tools to generate effective and durable conservation solutions for the benefit of wildlife and people. This paper is not a comprehensive review but rather a forward-looking and practical perspective that spans applications, taxa, regions and issues.

## Introduction

**Figure 1:**
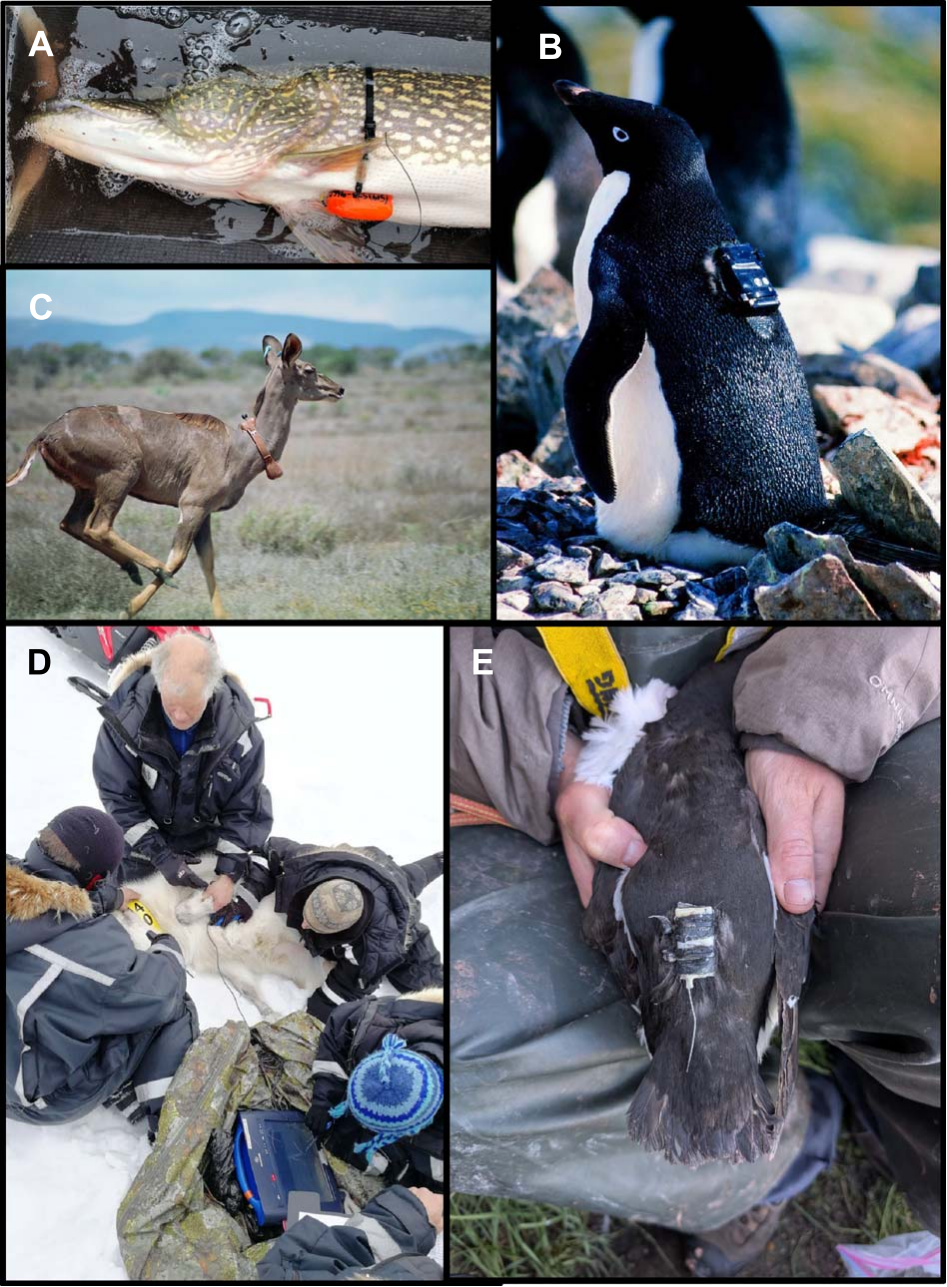
Images of biotelemetry and biologging in action in the service of conservation. (**A**) Northern pike (*Esox lucius*) affixed with an acceleration biologger for studying locomotor impairments arising from fisheries interactions (credit: Luc LaRochelle). (**B**) Adelie penguin (*Pygoscelis adeliae*) at Palmer Station in Antarctica in 1990 with a time/depth recorder that provided valuable data on foraging and food provision for chicks (credit: Mark Chappell). (**C**) Free-living kudu antelope (*T. strepsiceros*) affixed with biologgers to assess behavioural and autonomic responses to infection where bacterial pneumonia triggered sickness behaviour (a 60% reduction in activity detected by implanted loggers) and fever (core body temperature logger), accompanied by peripheral vasoconstriction (subcutaneous temperature logger) and selection of warmer microclimates (black globe temperature on collar) at fever onset (credit: Shane Maloney). (**D**) Data retrieval from subcutaneously implanted heart rate monitor in a wild Svalbard reindeer (*Rangifer tarandus platyrhynchus*) in Nordenskiöld Land, Svalbard, Norway. The animal is also instrumented with a GPS collar (Vertex PLUS, Vectronic Aerospace GmBH) containing an accelerometer (credit: L. Monica Trondrud). (**E**) Thick-billed murre (*U. lomvia*) with a Technosmart Marine AxyTrek GPS-accelerometer on its back, to classify behaviour and activity rate at Coats Island, Nunavut, Canada (credit: Jolie Nguyen).

Electronic tagging and tracking tools have revolutionized our understanding of the ecology of wild animals by allowing researchers to assess movement, habitat use and survival/mortality (Hussey *et al.*, 2015; Kays *et al.*, 2015; Wilmers *et al.*, 2015; Beltran *et al.*, 2025; Robichaud *et al.*, 2025). The toolbox continues to grow (Fig. 1) but is generally composed of two modalities—one that logs data until the device is retrieved and downloaded (known as biologging) and one that involves the transmission of signals that must be detected by a receiving system (biotelemetry; see Cooke *et al.*, 2021 for a discussion of the distinctions and similarities). There are also hybrid devices that log information and then transmit data as needed (e.g. pop-off satellite tags used on marine animals). In their most traditional form, such devices are used to position animals either through direct location of the tag (e.g. through triangulation, global positioning system; GPS) or the use of sensor information (usually light, pressure and temperature) to generate estimates of animal position using algorithms (most common in aquatic environments; see Musyl *et al.*, 2011). It is also possible to equip biologgers and biotelemetry devices with other sensors that tell us more about the animal than simply where it is (Cooke *et al.*, 2004). Indeed, it is this range of other sensors (reviewed in Whitford and Klimley, 2019) that tell us about the environmental conditions experienced by the animal or its physiological, energetic or behavioural state that is the focus of this paper. We recognize that the word biotelemetry in particular is broadly used to cover all types of animal tracking studies, and even in our paper at times we use examples that are focused more on location/movement data than on sensor data alone.

Animal tracking has been widely accepted as a means to generate actionable knowledge that can be applied to a range of challenges in conservation and resource management, from threat assessment (Cooke, 2008) to validation of conservation actions (Fraser *et al.*, 2018; Katzner and Arlettaz, 2020)—albeit see McGowan *et al.* (2017) for a more pessimistic perspective. Yet, considerations of how different forms of sensors that extend beyond the simple documentation of movement and habitat use are relatively uncommon despite their potential to provide a mechanistic understanding of conservation problems and solutions (Bogard *et al.*, 2010; Wilson *et al.*, 2015). Notably, when there have been efforts to explore and synthesize the use of sensors to address conservation issues, they are often specific to one electronic tagging modality (e.g. either biologging or biotelemetry) or to a specific group of taxa or realm (e.g. Lowerre-Barbieri *et al.*, 2019; Watanabe and Papastamatiou, 2023).

In this perspective article, we: (i) briefly summarize the history of biologging and biotelemetry where we emphasize the transition from fundamental to applied research, (ii) briefly consider the ethical aspects of using these tools, (iii) summarize the key ways in which these tools can be applied to support conservation and management to identify threats and evaluate actions and (iv) identify opportunities to further advance the ability of those tools to generate effective and durable conservation solutions for the benefit of wildlife and people. This paper is not a comprehensive review but rather a forward-looking and practical perspective. We posit that biologging and biotelemetry tools bridge behaviour and physiology to advance the conservation and management of wildlife—and have great potential to make important contributions in the coming decade. In this paper (including the title), we have embraced the word ‘bridge’ for the notion that behaviour and physiology are intertwined. Conceptually, they absolutely are, but when biotelemetry and biologging tools are used, it is often inference or correlations that are invoked rather than true ‘bridging’. For example, consider the studies that use accelerometers to quantify a behaviour (e.g. wing or tail beat) and then infer metabolic costs. As the array of potential sensors increases, we anticipate that there will be more studies that truly bridge behaviour and physiology through direct measurements of parameters that are specific to each of those domains. Overall, these tools have the potential to help achieve a more mechanistic approach to conservation (Cooke *et al.*, 2024), which is timely given the impacts of climate change and anthropogenic activities on wildlife (Ellis-Soto *et al.*, 2025).

## The history of biologging and biotelemetry

Biologging and biotelemetry technologies have been foundational to the remote monitoring and recording of animal behaviour and physiology in diverse environments. Biologging was the first to be introduced in the 1930s with pioneering work from Scholander (1940), who used a mechanical approach to assess the depth of marine mammals using capillary tubes. The earliest biotelemetry studies occurred between the 1950s and 1960s (see Hockersmith and Beeman, 2012 for review) where the movements of fish were monitored using acoustic telemetry (Trefethen, 1956), while radio telemetry was used to transmit physiological data about fish (Adams, 1965) and large terrestrial mammals (Bligh and Harthoorn, 1965) and to track the movements of terrestrial animals (LeMunyan *et al.*, 1959; Cochran and Lord, 1963). Most of the early devices were custom devices (as opposed to the many commercially available devices that exist today), developed in partnership with electronics experts and biologists. An example of that early creativity is the work on bears where various sensors were incorporated into radio transmitters (see Folk Jr, 1964) that allowed the research team to measure the electrocardiogram during hibernation to understand human impacts on free-living wildlife (Folk Jr *et al.*, 1972). Throughout the last five decades, there have been advancements in biologging and biotelemetry technologies with the miniaturization of the technology (Lennox *et al.*, 2025), better battery efficiency, increased data capacity, improved transmission and positioning capabilities and the addition of more complex and diverse sensors, which have often been translated from the human medical realm (Evans *et al.*, 2013; Laske *et al.*, 2021). The miniaturization of these technologies opened new possibilities to monitor smaller animals across a range of taxa (Shillinger *et al.*, 2012). Advancements in battery life (e.g. use of lithium) and increased storage capacity have created opportunities to collect data on the same individuals across seasonal, annual and ontogenetic changes (Block *et al.*, 2011). Because animals can move long distances depending on their life-history strategies, being able to transmit data is important for the recovery of those data. Improvements in satellite technology have allowed for the continuous transmission of data, or in the case of archival data, delayed transmission when the tags pop-off the animal or when an animal surfaces (Ryan *et al.*, 2004; Evans *et al.*, 2013). GPS has been important for terrestrial animal research, allowing animal locations to be measured nearly continuously (Wikelski *et al.*, 2007; Bridge *et al.*, 2011). Similarly, the positioning of animals in aquatic environments can be determined using the triangulation of acoustic detections (e.g. Guzzo *et al.*, 2018). Finally, the development of sensors that are available for both biologging and biotelemetry tags has advanced substantially and has improved our understanding of the behaviour (e.g. migration, spawning, feeding) and physiology (e.g. metabolism, heart rate, sleeping patterns) of animals in the wild (reviewed in Whitford and Klimley, 2019).

Biologging and biotelemetry were initially designed to study fundamental behaviour and physiology in free-ranging animals, but they have also had substantial use in applied research (Bogard *et al.*, 2010). These tools yield valuable insight into animals outside the laboratory, providing realism and the ability to link physiological state with the environment, thus contextualizing behaviours (Hussey *et al.*, 2015; Kays *et al.*, 2015). The technologies have been useful for wildlife and habitat management (Wilson *et al.*, 2015; Brownscombe *et al.*, 2022) and policy making in conservation (Heylen and Nachtsheim, 2018). For example, these technologies have been used to mitigate negative human–animal conflicts (Bastille-Rousseau *et al.*, 2018), for threat assessment of imperilled species (see Cooke, 2008) and to identify and protect key reproduction sites (Brownscombe *et al.*, 2019b). Beyond an assessment of the state and behaviour of animals, data from different sensors can be used to collect environmental data (see Boehlert *et al.*, 2001). Together, biologging and biotelemetry have been valuable to answer fundamental questions about animals, to collect novel data about the environment, as well as for the management and conservation of wild animals.

## Ethics of biologging and biotelemetry tagging and tracking

Underpinning the ability to use biologging and biotelemetry in animal conservation is the need for ethical tagging methods that maintain the welfare of tagged individuals (McIntyre, 2015). Such requirements are not just about adherence to ethical standards (and regulations in many cases) for the tagged individuals, but also to ensure that the social licence to do such research is maintained and that the data generated are representative of untagged conspecifics. It is important to acknowledge that individual animals affixed or implanted with electronic tags are not better off for it, although every effort is made to minimize the associated cost. The population as a whole may benefit from knowledge that is derived from the study of an individual, but that tagged individual is likely to not benefit and has the burden of being handled, undergoing any related procedures (e.g. surgery) and carrying the device. There may be some instances where the costs of tagging borne on the individual do not outweigh the benefits derived from the generation of new knowledge (Cooke *et al.*, 2013) but fortunately there have been many refinements to procedures for handling and tagging as well as reductions in device size that collectively benefit a diverse range of taxa through the application of ‘best practices’ (Hawkins, 2004; Geen *et al.*, 2019; Horning *et al.*, 2019; Manville *et al.*, 2024). Importantly, refinements and learnings are not static, and as the evidence base evolves, so does best practice. One way to ensure that good practice has been applied and to ensure potential for assessment and learning by readers is to ensure that papers embrace minimum reporting standards that include essential information to understand what was done and why, in terms of tagging methods (Thiem *et al.*, 2011; Clemens *et al.*, 2023; Payne *et al.*, 2026). The sharing of data via repositories like Movebank and Ocean Tracking Network creates opportunities to mine existing data, which could reduce the need to tag additional animals and is an example of the ‘reduce’ aspect of the three ‘R’s’ in animal welfare.

When tagging methods impact the long-term welfare of animals or the animals fail to recover from the tagging processes, data quality is severely impacted. A tenet of biologging studies is that the behaviour, condition and fate of tagged individuals is similar to that of untagged conspecifics (Brown *et al.*, 2011). Consider cases where tagged individuals select a different habitat from untagged animals, which would lead to erroneous conclusions about critical habitats, or if animals never recovered fully from tagging such that measures of subsequent responses to anthropogenic stressors were altered relative to what they would be in untagged conspecifics. Given the applied nature of conservation physiology, the data derived from animals that are not representative of their untagged conspecifics could mislead conservation decision-makers and potentially harm wildlife populations. Moreover, if the public encounters animals that have been tagged and are doing poorly, then the social licence to do such work can be impeded (Hammerschlag *et al.*, 2014). Overall, it is the responsibility of researchers and their institutions to ensure that the methods used to tag and track wildlife with biotelemetry and biologging devices are optimized to ensure that the welfare of tagged individuals is maintained. Researchers should continually strive to generate evidence to guide them with their methods and share their learnings with others.

Beyond the impact that can occur to the individual animal as a result of the tagging procedures or tag burden, it is also important to consider other unintended outcomes that may be deleterious to tagged animals (or their untagged conspecifics; Cooke *et al.*, 2017). For example, data on the biology of imperilled or valuable species could be used to enable exploitation (legal or illegal). Given the push for open data, it is possible for members of the public to figure out where animals are and what they are doing. In extreme cases, there are examples of ‘cyber hacking’ where individuals seek to gain access to otherwise protected tracking data. Although a full treatment of these issues is beyond the scope of this paper (see Cooke *et al.*, 2017 for a more complete summary), it is important for those who use biologging and biotelemetry tools to make sure that their work does more good than harm. In some cases, the best approach may come by not doing a study at all if there is a strong likelihood that the data could be used for malicious purposes.

## Applied biologging and biotelemetry—relevance to understanding threats

Biologging and biotelemetry show much promise to help understand the threats that face wildlife. Here we briefly discuss key threat categories [as per the International Union for the Conservation of Nature/ Conservation Measures Partnership (IUCN/CMP) Threat Classification V4.0; IUCN—CMP Direct Threats Classification Version 4.0—conservationstandards.org] and the various ways in which biologging and biotelemetry have been applied to understand and document those threats. This threat classification is considered ‘the’ global standard and provides a common language to assess and categorize threats. It was developed after extensive consultation with scientists and practitioners from around the globe. The threat categories are updated regularly (we used Version 4.0) reflecting the opportunity for the standards to evolve based on emerging threats or relevant feedback from users. We draw upon examples that span different taxa and regions, where possible.

### Residential, commercial and recreation areas

As human settlements continue to encroach on the wild spaces that remain on Earth, across both terrestrial and aquatic ecosystems, biologging and biotelemetry have become crucial to reveal how animals respond to mounting anthropogenic stressors (Cooke *et al.*, 2024). First, biologgers and biotelemetry can both show how animals change their movements, behaviour and habitat use in these altered environments, which itself can be indicative of individual responses to stressors. For instance, biotelemetry revealed that recreation infrastructure was avoided both during the summer (e.g. mountain biking trails) and winter (e.g. ski pistes) by western capercaillie (*Tetra urogallus*) in Germany (Coppes *et al.*, 2017). Next, biologging and biotelemetry can also capture the physiological and energetic consequences of these interactions with humans and their infrastructure (Wilson *et al.*, 2015). Heart-rate loggers showed that exposure to people engaged in recreation (e.g. pedestrians and equestrians) caused significant increases in heart rate in two ungulate species (Reimoser, 2012). In addition, many biologging and biotelemetry devices incorporate environmental sensors, such as temperature, dissolved oxygen, salinity, pressure or light sensors, that allow researchers to link habitat conditions to animal behaviour and physiology. For example, light loggers revealed that artificial illumination in urban areas of Munich, Germany increased perceived day length in European blackbirds (*Turdus merula*; Dominoni and Partecke, 2015). Taken together, these examples highlight how biologging and biotelemetry can reveal the consequences of residential, commercial and recreational areas for various taxa.

### Agriculture and aquaculture

In the last 20 years, biologging has become integral to the measurement of behavioural and physiological data in many animal production systems. The farmers who use the technology, and the developers who create it, term the technology ‘Precision Farming’. Researchers often use technology that has been developed for production animals, which once validated (see Niccolai *et al.*, 2024 for an example), can be readily applied to other species, often at reduced cost. Particularly promising for wildlife research are new sensors that have been developed in the agriculture sector for measurements of jaw movement (to assess feed intake and rumination activity), rumen pH, reticular contractions, electrical resistance of the vaginal mucus (which can be used to predict ovulation), biological components (enzymes, antibodies or microorganisms), odour, glucose, progesterone and individual milk components (Dzermeikaitė *et al.*, 2023). In aquaculture, electronic tags with sensors that monitor feeding behaviour of captive fish have been used to optimize feeding such that there is less food waste released into the environment (Macaulay *et al.*, 2021). In most parts of the world, agricultural activities lead to dramatic changes in landscapes. Biologging can thus be a useful tool to assess potential effects on wildlife. The use of accelerometer biologgers affixed to European hare (*Lepus europaeus*) in an agriculture-dominated landscape revealed that hares that occupy more diverse habitat (i.e. numerous smaller farm fields interspersed with natural areas) were able to spend more time resting and were more efficient at food acquisition relative to those in larger, more homogeneous farm fields (Ullmann *et al.*, 2023). In general, there is much opportunity to further apply biologging and biotelemetry tools to aquaculture and agricultural systems to understand potential impacts while also embracing novel sensors that emerge from the precision farming sector.

### Energy production and mining

Although accelerometers, GPS, temperature sensors and time depth recorders have been used to study the movement behaviours of animals (e.g. Raya Rey *et al.*, 2010), these sensors are still underutilized to assess the responses of wild animals to the human activities that are associated with energy generation and mining. The use of collars with accelerometers and GPS revealed that mining disturbance caused northern quolls (*Dasyurus hallucatus)* to select fragmented, rocky and disturbed habitats, which were associated with the highest overall activity rate and energy costs (as reflected by vectorial sum dynamic body acceleration; VeDBA) (Cowan *et al.*, 2024). GPS-loggers with barometric pressure sensors revealed that the flight height of northern gannets (*Morus bassanus*) during active foraging put these birds at a higher risk of collision with wind turbines than did their flight height during commuting (Cleasby *et al.*, 2015). Turbulence around wind farms has also been shown to increase the metabolic costs of animal flight (or cause them to fly further to avoid turbulence), especially for animals that are small and flap their wings while flying (Shepard, 2025). In aquatic environments, seabirds are at high risk of collisions with the moving parts of underwater tidal energy devices (Isaksson *et al.*, 2020). GPS tags with time depth recorders have been useful tools to determine how dive metrics, such as depth and duration, change in response to tidal turbines (Isaksson *et al.*, 2021). Solar farms change the microhabitat and microclimate of environments, with consequences for animal behaviour, physiology and survival (Brand *et al.*, 2016; Chock *et al.*, 2020). While studies have found direct mortality risk from collision or solar flux (Chock *et al.*, 2020), thermal loggers attached to animals have shown that animals can also utilize the areas under solar panels for thermoregulation based on thermal loggers attached to animals (Fonseca *et al.*, 2023).

The construction of renewable energy technologies itself can affect the physiology of animals. Cones *et al.* (2024) studied the impacts of pile driving during the construction of offshore wind turbines on the giant sea scallop (*Placopecten magellanicus*) by combining on-board measures of tri-axial acceleration and magnetic field strength, with fibre-optic oxygen probes *in situ*. They found that scallops exposed to the noise and vibration of pile driving had reduced valve openings and lower oxygen levels in mantle water, which had downstream negative consequences for metabolic rate and antipredator behaviours (Cones *et al.*, 2024). Finally, it is also becoming increasingly clear that the infrasound associated with wind turbines and other anthropogenic noise has negative effects on the cardiovascular and neurological health of humans (Kapoor *et al.*, 2025). Biologging tools that measure health indicators in wild animals would be a much-needed approach to monitor animal responses to the chronic noise and vibration of energy generation.

### Transportation, service and security corridors

As humans continue to alter landscapes in water, on land and in space, biologging and biotelemetry have been useful to understand threats related to roads, railroads, air traffic, service lines, shipping lanes, space activities, fencing and walls, among other transportation and service or security corridors. These anthropogenic threats can affect different parts of a species’ biology and ecology, including their movements or migrations, their foraging behaviour and their physiology. Even recreational drones, which are seemingly non-intrusive, can impact animal behaviour and stress responses. For instance, through the use of biologgers and GPS collars, Ditmer *et al.* (2015) showed that black bears (*Ursus americanus*) exhibited substantially elevated heart rates when disturbed by drones, although they only infrequently moved away from the disturbance. The same researchers later showed that black bears had a physiological response to road crossings, albeit a much smaller increase in heart rate, even when the bears did not cross the road (Ditmer *et al.*, 2017). Weisenberger *et al.* (1996) investigated how noise from low-altitude jet aircraft affected the behaviour and heart rate of desert mule deer (*Odocoileus hemionus crooki*) and mountain sheep (*Ovis canadensis mexicana*) in captivity and found that heart rate increased with noise levels, but returned to baseline levels within 1 to 3 min. Grey seals (*Halichoerus grypus*) equipped with long-duration audio and 3D-movement tags were shown to be exposed to sound from ship traffic for 2–21% of their time in water and that the noise often interrupted behaviours like resting (Mikkelsen *et al.*, 2019). Seismic airguns and ship noise in the Arctic altered cardiovascular, respiratory and locomotor reactions of narwhals (*Monodon monoceros*), as shown by heart rate-accelerometer-depth recorders and doubled the energetic cost of diving (Williams *et al.*, 2022). These examples show but a few of the ways in which biologging can be used to assess and better understand the consequences of human transportation on wildlife. Nevertheless, there is much room to expand on this research as mammalian studies dominate this field, yet all taxa are presumably affected by human transportation and related services.

### Biological resource use and control

Biologging and biotelemetry have much to offer with respect to understanding threats related to biological resource use and control. Although harvest is largely about life-tables and population dynamics, the reality is that the use of biological resources often has collateral damage that can result in negative impacts on non-target species at the individual level and potentially scale up to impact populations. For example, the harvest of forest resources has been predicted to have negative impacts on the thermoregulatory behaviour and physiology of understory birds in Amazonia, as assessed using small thermal loggers in harnesses on nine species (Jirinec, 2024). For freshwater turtles encountered as bycatch in a commercial hoop net fishery, biologgers equipped with acceleration, temperature and depth sensors revealed behavioural impairments that lasted for several hours, although the animals were fully recovered by 48 h after the encounter (Gutowsky *et al.*, 2017). When moose (*Alces alces*) are hunted with dogs, Graesli *et al.* (2020) used subcutaneous heart rate loggers, ruminal temperature radio transmitters and GPS collars equipped with acceleration sensors, to show that when the dogs were within 240 m for >10 min there was an increase in body temperature and heart rate in the moose for ~24 h on the day of disturbance, and that the moose rested for ~ 90 min or more on the subsequent day than they did prior to the disturbance (Graesli *et al.*, 2020). These examples collectively illustrate that the use of biological resources via activities such as forestry, hunting and fishing can have negative sublethal biological impacts on animals (even if they are not harvested) with unclear long-term fitness consequences.

### Human intrusions and disturbances

Human intrusions and disturbances into natural areas come in many forms and have the potential to impact wildlife in diverse ways. One of the potentially more extreme examples comes from military activity and warfare (reviewed in Lawrence *et al.*, 2015). Given the inherent danger to researchers when those activities are studied *in situ*, biologgers have been applied for such work. Krausman *et al.* (1998) conducted one of the first studies with heart rate loggers that revealed that mountain sheep (*Ovis canadensis nelsoni*) experienced transient increases in heart rate when they were exposed to military jets flying overhead. A more recent example of a ‘real’ conflict involved the use of accelerometers to assess how snakes (*Spalerosophis diadema*) responded to explosions from missiles launched by another country and associated missile defence actions (Liang *et al.*, 2026). That study revealed that snakes exhibited immediate movement behaviours as they sought cover during attacks. Disturbance can also occur from what might seem comparatively benign activities such as ecotourism. Naylor *et al.* (2009) equipped North American elk (*Cervus canadensis*) with motion-sensing radio tags and revealed that off-road recreational activities including all terrain vehicles (ATVs), mountain biking, horseback riding and hiking all induced alterations in behaviour. Marchand *et al.* (2025) studied the potential disturbance of Alpine ibex (*Capra ibex*) tagged with GPS collars (that contained accelerometers) to trail running events as well as several legs of the Tour de France in the French Alps. Trail running yielded more behavioural alterations than did the cycling event, although that was presumably a function of seasonal differences in animal distribution and that the cycling event was limited to the summer period. Heart rate and body temperature loggers revealed a strong physiological response of free-ranging greylag geese (*Anser anser*) to re-occurring fireworks, with the increase in body temperature of the geese directly following New Year’s Eve fireworks being indicative of stress and an increase in energy costs (Wascher *et al.*, 2022). Taken together, these studies highlight important and often overlooked impacts of human activities on wildlife. Such information can aid management decisions to restrict or redirect intrusions during vulnerable periods, such as reproduction and migration.

### Natural system management and modifications

Direct and indirect ever-increasing anthropogenic impacts continue to reduce the amount of space available to wild animals and modify the environment in protected areas that are set aside for wildlife. The changes can affect critical ecosystem functions, such as migration (Kauffman *et al.*, 2021). To address possible resource limitation, conservation managers and tourism operators can modify natural systems through resource management, such as the provision of artificial water sources (Edwards *et al.*, 2017) and supplementary food (Newey *et al.*, 2009). These measures are intended to conserve healthy ecosystems, but modifications can have unforeseen repercussions (Smit *et al.*, 2007). The use of biologgers to record animal movement, physiology and other parameters can provide insight into individual performance and the impact of the interventions and thereby document their success (or not) and inform conservation actions (Ellis-Soto *et al.*, 2025). Core body temperature can be used to determine whether animals experience resource limitation, as reported in large herbivores (Hetem *et al.*, 2016), where such physiological measurements could identify periods when animals would benefit from interventions, such as the provision of water, shade or supplementary forage and quantify the effect of such interventions. Taking advantage of natural behaviour, animal-borne sensors can be used to monitor changing environmental conditions, as evidenced by the documentation of oceanographic data from biologgers that are attached to the backs of foraging marine birds (Orben *et al.*, 2025) and the measurement of lake temperatures from implanted loggers in freshwater fish (Kraus *et al.*, 2022).

### Invasive/other problematic species, genes and pathogens

Ecosystem structure and function may be altered by invasive, non-native (including domesticated) and problematic native species, as well as by pathogens or transduced genetic material within a population. Integration of physiology into invasion biology has been recognized as a critical priority to develop strategies that minimize invasion risks and mitigate their impact (Boardman *et al.*, 2022). Biologging and telemetry technologies enable researchers to assess physiological responses in individuals, identify shifts in behaviour and species interactions and evaluate potential ecosystem-level consequences (Lennox *et al.*, 2016). For example, body temperature biologging combined with movement tracking in Burmese pythons (*Python bivittatus*) revealed that this invasive species was unlikely to extend its range into more temperate regions because of cold-induced impairments (Dorcas *et al.*, 2011), whereas acoustic telemetry used to monitor activity and depth patterns in wild lionfish (*Pterois miles*) demonstrated that high individual-level variability likely contributes to the species’ success in its invasion of the Mediterranean Sea (Gavriel *et al.*, 2021). Biologging can also detect behavioural and autonomic responses to infection, as shown in free-living kudu antelope (*Tragelaphus strepsiceros*), where bacterial pneumonia triggered sickness behaviour (a 60% reduction in activity detected by implanted loggers) and fever (detected by a core body temperature logger), accompanied by peripheral vasoconstriction (detected by a subcutaneous temperature logger) and selection of warmer microclimates (assessed via changes in black globe temperature on a collar) at fever onset (Hetem *et al.*, 2008). Biologging technology also revealed the consequences of infection for febrile monkeys (*Chlorocebus pygerythrus*). Monkeys with fever continued to engage in social behaviour (with potential facilitation of the spread of a pathogen) but were more likely to be targeted with aggression by their conspecifics and to become injured, compared to afebrile monkeys (McFarland *et al.*, 2021). By providing insights on outbreaks of infection, animal physiology, behaviour and subsequent health and mortality, biologging-based disease surveillance can inform management decisions, support disease control measures and advance wildlife conservation (Talmon *et al.*, 2025). Nevertheless, despite its potential to reveal welfare impacts, physiological biologging remains underutilized compared to spatial tracking to understand the effects of invasive species, other problematic species and disease.

### Pollution

The IUCN lists pollution as one of the top threats to global biodiversity. Biologgers that include GPS-accelerometers and heart rate loggers have provided valuable insights into the effect of pollutants such as chemicals, light and noise on wildlife. In terms of chemical pollutants, biologging has revealed complex interactions among pollutants, hormones and behaviour. GPS-accelerometers, for example, revealed that shorter dive durations of thick-billed murres (*Uria lomvia*) were correlated with higher circulating triiodothyronine (T_3_) thyroid hormone concentrations, which are positively associated with resting metabolic rate in murres (Elliott *et al.*, 2013; Esparza *et al.*, 2022). However, the relationship between T_3_ and time spent diving was not found in years of early ice break-up in murres with high concentrations of methyl mercury (MeHg) in their blood. This was possibly because the early break-up of the sea ice increased stress levels in murres, while the effects of MeHg on T_3_ interfered with the murres’ ability to modulate their underwater foraging time (Esparza *et al.*, 2022). The effects of human disturbance via artificial light at night (ALAN) on several wildlife species, including lobsters, fish, birds and hedgehogs, have been revealed by biologging. The use of heart rate loggers paired with tri-axial accelerometers showed that low levels of ALAN had no effect on the heart rate or locomotion of Caribbean spiny lobster (*Panulirus argus*) (Steell *et al.*, 2020). In contrast, activity levels, as estimated by overall dynamic body acceleration using accelerometers, were higher in smallmouth bass (*Micropterus dolomieu*) that were continuously or intermittently exposed to artificial light in comparison to control fish (Foster *et al.*, 2016). Finally, European hedgehogs (*Erinaceus europaeus*) displayed greater activity and movement in areas with lower levels of ALAN (Berger *et al.*, 2020). In addition to the examples given above for transportation and energy generation, the impacts of noise pollution have been tested using biologgers and biotelemetry in several other species, including fish, turtles and marine mammals (see also examples under ‘Human Intrusions & Disturbances’). In response to higher levels of noise pollution from boating activity, mulloway (*Agyrosomus japonicas*) reduced their activity levels and inhabited greater depths, as revealed by acoustic accelerometry (Payne *et al.*, 2015). Custom-built multichannel data loggers revealed that greater exposure to noise pollution altered the activity-specific time budgets of green turtles (*Chelonia mydas*), causing them to spend greater time travelling, scanning and remaining on the seafloor (Diaz *et al.*, 2024). Acoustic accelerometers revealed that an increase in anthropogenic noise led to increases in the foraging effort of killer whales (*Orcinus orca*; Tennessen *et al.*, 2024). Collectively, these examples reveal the many ways in which biologging and biotelemetry can be used to assess threats related to different classes of pollutants.

### Natural disasters

Natural disasters are widely recognized for their negative impacts on human health and well-being along with extensive socio-economic costs but also have the potential to alter the behaviour and physiology of wildlife. Much has been written about the potential of biologging and biotelemetry tools to opportunistically study the consequences of natural disasters ranging from wildfires to hurricanes to earthquakes (and more) on wildlife (see Lennox *et al.*, 2023; Beltran *et al.*, 2025). Yet, examples of such opportunistic studies, aside from those that solely assess changes in animal space use (Gutowsky *et al.*, 2021) are rare. One of the most compelling studies involved short-term earthquake forecasting using the monitoring of farm animals (i.e. dogs, cows, sheep) in Italy with acceleration sensors on biologgers (Wikelski *et al.*, 2020). Over several years, thousands of earthquakes occurred with a wide range of intensity, and animals exhibited consistent anticipatory responses indicated by increases in locomotor activity. Interest in the prediction of natural disasters through the monitoring of animal behaviour continues to grow (see Wason *et al.*, 2025). Another example comes from hurricanes, where Crowe *et al.* (2020) revealed that loggerhead sea turtles (*Caretta caretta*) tagged with depth- and temperature-sensing satellite transmitters, which did not flee the primary hurricane path, spent more time engaged in dives. These individuals were subsequently exposed to cooler sea surface temperatures as well as a deeper thermocline, which presumably had bioenergetic consequences. For example, via the use of temperature sensors implanted in the brown antechinus (*Antechinus stuartii*), Stawski *et al.* (2015) showed that torpor use increased following a forest fire, thus reducing energy requirements and predator exposure, a response which may be key to survival as the frequency and intensity of wildfires increases (Moritz *et al.*, 2014). As more wildlife are equipped with biologging and biotelemetry tags, we anticipate more opportunistic studies that reveal the true costs of diverse forms of natural disasters on wildlife.

### Climate change

Across the globe, the rate of climate change is occurring faster than the rate at which many animals can respond genetically. In addition to long-term changes in environmental regimes, the frequency and severity of extreme weather events is increasing (IPCC, 2023). Biologging and biotelemetry tools allow us to investigate both short- and long-term physiological and behavioural responses to climate change under natural conditions (Chmura *et al.*, 2018). In the Arctic, where cold-adapted animals may particularly struggle with warming temperatures and heat waves, subcutaneous body temperature, heart rate and accelerometer biologgers in reindeer (*Rangifer tarandus*) revealed that a severe heat wave led to shifts in diel activity patterns, lowered heart rates and increased body temperature, with negative consequences for body condition in the autumn (Trondrud *et al.*, 2023). Biologgers that measure heart rate and core body temperature have also been used to show that cold-adapted common eiders (*Somateria mollissima*) had to make more frequent stops to lower their body temperatures during their spring migration, to prevent overheating (Guillmette *et al.*, 2017). In savannas, collar temperature loggers and ingested temperature loggers have revealed the importance of shuttling to water sources, and the availability of water, for African elephants to thermoregulate (Mole *et al.*, 2018; Thaker *et al.*, 2019). Temperature and activity biologging in aardvarks (*Orycteropus afer**)*** and ground pangolins (*Smutsia temminckii)* have shown that these dryland mammals are susceptible to starvation as a result of increasing droughts with climate change (Weyer *et al.*, 2020; Panaino *et al.*, 2023). Biologging technology can also uncover long-term responses in energetic costs and phenological shifts. For instance, implanted temperature loggers in Arctic ground squirrels (*Urocitellus parryii*) showed that ‘thermogenic torpor’ (that prevents the animal from freezing when it is torpid) was not required as often at the now more common higher soil temperatures, which led to a reduction in energy expenditure during hibernation and an increase in body mass in females during the spring (Chmura *et al.*, 2023). The authors documented that over 25 years, spring emergence has advanced by 14 days for females, but not males, a mismatch that may negatively impact population dynamics. As climate change imposes novel and more demanding energetic challenges for wildlife, biologging can enable us to understand the benefits and costs of physiological and behavioural plasticity in a changing world.

### Unknown threats

The ‘unknown threats’ category is inherently difficult to characterize because they are, by definition, ‘unknown’. However, because electronic tags equipped with sensors tell us about how organisms perceive, use or otherwise interact with their environment, such devices are well-positioned to identify unknown (or unstudied) threats or stressors to determine if and how they are relevant to wildlife fitness and population persistence. Indeed, many of the threats that we have identified above were ‘unknown’ at one point, so perhaps this classification is also about ‘emerging threats’. The interaction of multiple threats may produce unanticipated consequences that would also be relevant to this classification. For example, climate change is a threat multiplier (Goodman *et al.*, 2023) so may interact in complex and unanticipated ways with other threats.

## Applied biologging and biotelemetry—relevance to conservation actions

Here we use the IUCN/CMP Conservation Action categories (V4.0—see https://docs.google.com/spreadsheets/d/1i25GTaEA80HwMvsTiYkdOoXRPWiVPZ5l6KioWx9g2zM/edit?gid=1144804238#gid=1144804238) to provide an overview of the potential ways in which biologging and biotelemetry are relevant to conservation actions. Several actions (e.g. *Livelihood, Economic and Moral Incentives; Legal and Policy Frameworks*) are not as obviously relevant and were therefore excluded. However, we did envision some connections with those actions. For example, for *Livelihood, Economic and Moral Incentives,* issues that are related to sustainable tourism come to mind. For *Legal and Policy Frameworks*, there are certainly opportunities for ‘creating, amending, or influencing policies and guidelines at all levels’, which would arguably be informed by *Research and Monitoring* (which is covered in full below).

### Land/water management

Biologging and biotelemetry techniques are becoming important tools for conservation management of both land and water habitats. Because animals are highly mobile and often hard to observe, the attachment of biologging technology provides the ability to identify specific locations that animals use to increase their fitness, whether that be foraging areas (Sawyer *et al.*, 2009) or reproductive sites (Nemeth *et al.*, 2023). The use of biologgers and biotelemetry to gather data on specific fitness-related activities (e.g. foraging, migration, spawning or rest) that can be paired to habitat, managers are able to identify key areas that may need conservation actions both spatially and temporally. More common use of these technologies for management of land and water is an assessment of how animals use protected areas (Wilson *et al.*, 2008b; Guixe and Arroyo, 2011; Lea *et al.*, 2016; Hofmann *et al.*, 2021; Nemeth *et al.*, 2023), which can be used to provide information that enhances their efficacy (e.g. timing, size, area). Advances in biologging allow for fine-scale behavioural segmentation and direct identification of physiological states that are critical to survival, such as electrophysiological sleep in wild mammals and birds (Kendall-Bar *et al.*, 2022). For animals whose access to safe sleep depends on their physiological state, such as the northern elephant seal (*Mirounga agustirosis*), the management of habitats where animals can engage in safe and quality rest or sleep is important (Kendall-Bar, 2023; Kendall-Bar *et al.*, 2023). These tools enable evidence-based spatial and temporal data that can be used for the management of habitats and protected areas for aquatic and terrestrial habitats that are specific to fitness-gaining activities in animals.

### Species management

Once used primarily to address fundamental ecological questions (Donaldson *et al.*, 2014), biologging and biotelemetry are now instrumental tools in species management (Crossin *et al.*, 2017; Hinch *et al.*, 2025). In terms of movement and behaviour, these technologies can identify critical habitats (White *et al.*, 2024; Piczak and Chow-Fraser, 2019), guide the design of protected areas (Lea *et al.*, 2016; Doherty *et al.*, 2017) and inform threat mitigation strategies (Aschettino *et al.*, 2020; Gulsby *et al.*, 2011; Piczak *et al.*, 2019). Environmental sensors that are integrated into biologging and biotelemetry devices can aid species management via the collection of fine-scale environmental data such as water temperature (Lennox *et al.*, 2018), black globe temperature (Hetem *et al.*, 2007) or water conductivity (Blanchet *et al.*, 2015), with applications that include the identification of habitat conditions that support survival and the detection of emerging environmental stressors. As spatial aspects of biologging and biotelemetry have been incorporated into species management, the next frontier is arguably conservation physiology, with measurements of physiological stress through technologies such as heart rate loggers. Via the integration of spatial movements, environmental conditions, behavioural and physiological responses, biologging and biotelemetry offer managers a powerful suite of tools to develop targeted and effective species management strategies.

### Conservation designation and planning

As anthropogenic pressures on natural spaces increase, it is crucial to identify key locations for conservation, which may encompass seasonal home ranges and movement corridors. Biologging can reveal areas where animals engage in particular behaviours, such as feeding sites for juvenile green turtles (*C. mydas*) in the Bahamas, where recommendations were made for increased protection for those locations (Fuentes *et al.*, 2019). Likewise, biologgers in grey reef sharks (*Carcharihinus amblyrhinchos*) were used to identify a super-habitat used for breeding, foraging and shelter that should be incorporated into marine conservation plans (Papastamatiou *et al.*, 2025). Wide sampling within a population can be critical, since not all individuals will make use of the same locations, as is the case for red-footed boobies (*Sula sula*) in the Seychelles, and a comprehensive understanding of species needs is necessary to inform conservation planning (Nicoll *et al.*, 2025). As indicated above, efforts to increase the use of sustainable energy, such as offshore wind farms, can have detrimental effects on some species, such as migrating birds. Transport planning to reduce ship strike risk has also been informed by use of biologgers. Chapple *et al.* (2024) used camera and inertia data-loggers on basking sharks (*Cetorhinus maximus*) to yield information needed to develop vessel restrictions. Biologging and biotelemetry data can provide detailed information on the timing and extent of such impacts, which can then be translated into conservation actions (Schwemmer *et al.*, 2023).

### Awareness raising

Because biologging and biotelemetry use cutting-edge technology (Chen *et al.*, 2026) and have the potential to reveal mysteries of the natural world (Pacheco, 2018), there is potential to tell stories that engage diverse public and raise awareness for the plight of wildlife. Published examples are rare (despite calls for McGowan *et al.*, 2017 to do so), although many scientists routinely engage in various forms of science communication. Popular media outlets can often raise awareness by sharing captivating stories and photos. A fantastic example is from 1971, where a photo of a gentoo penguin equipped with a ‘vest’ that housed various environmental and physiological samples adorned the pages of National Geographic Magazine (https://www.nationalgeographic.com/photo-of-the-day/photo/penguin-antarctica-biology-backpack). More recently, several animals equipped with biotelemetry devices have active social media presences including Lydia the Great White Shark who carries a fast-lock GPS transmitter with a temperature sensor (see news article: https://www.cbc.ca/news/science/great-white-shark-secrets-revealed-by-tracking-lydia-s-tweets-1.2984242). Several scientist artisans (i.e. Jaimie Cleeland and Annalise Rees) in Tasmania created a jewellery brand that is based on the use of recovered loggers (from birds) as a way to start conversations and raise funds for conversation (see news article; https://www.abc.net.au/news/2016-09-14/geolocation-journeys-bird-brooches-help-science-research/7840138). A team in Canada created an educational programme called ‘AquaTrax’ that developed curriculum-relevant content so that teachers could use tracking science studies in their classroom learning (see some materials here; https://www.youtube.com/channel/UCtcY6YRroUcRMyz_rugaDiA). Clearly, there is more space for creativity in the telling of our stories to generate public awareness and action related to wildlife conservation. However, that needs to be tempered with the reality that because some aspects of biologging and biotelemetry are somewhat invasive, the sharing of stories can also elicit public concern for animal welfare.

### Law enforcement and prosecution

Biologging and biotelemetry have the potential to contribute to law enforcement and prosecution. The most obvious way is through the use of anti-poaching tags that report information that can detect an animal that has been injured or killed (along with the location) such that enforcement staff could be deployed to determine if illegal activity was involved (O'Donoghue and Rutz, 2015). In an application of this approach, Jiguet *et al.* (2023) used satellite tags equipped with thermal and acceleration sensors that had been obtained from tagged Eurasian Curlew (*Numenius arquata*), an imperilled shorebird, to identify the exact timing and location of illegal kills. The use of sensors helps to move from inferred poaching based on general changes in location to more subtle measures of locomotor activity or thermal biology that provide more evidence regarding likely outcomes. Acceleration sensors interfaced to a wireless network have been deployed on fences at wildlife reserves to detect potential security breaches (Wittenburg *et al.*, 2007). In general, the logical application of electronic tagging and tracking tools for enforcement or deterrence is through the use of hidden tags that would allow enforcement staff to go directly to the poacher before poaching had occurred (based on evidence that the animal was actively being pursued by poachers) or after the harvest had occurred.

### Research and monitoring

A basic understanding of a system is fundamental for conservation practices to be applied effectively. Biologging tools provide the unique opportunity to collect baseline data on species physiology and behaviour, which can provide the necessary information on how and when conservation should take place (see examples under ‘Species Management’ and ‘Conservation Designation & Planning’), as well as the effectiveness of specific actions. In the cases of reintroduction and translocation for conservation purposes, monitoring animals pre- and post-release is essential to evaluate the impact of these actions on the species and individuals (Tarszisz *et al.*, 2014). For example, baseline information from biologging on the physiology of scimitar-horned oryx (*Oryx dammah*), a once-extinct species that is currently being reintroduced to the wild, allows for the identification of potential stressors that may affect their capacity to adapt in the wild (Leimgruber *et al.*, 2023). Such information is important to assess and improve conservation practices and the management of critically endangered species. While translocations and reintroductions aim to restore species to their native ranges or restore ecosystem functions, for the translocated individual, release into a novel environment can be a very stressful event (Dickens *et al.*, 2010). These changes may also alter the behaviour of the translocated individual post-release. Eline *et al.* (2023) put accelerometers on reintroduced and translocated New England cottontails (*Sylvilagus transitionalis*) and found that they tended to spend more time vigilant and moving, making them potentially more vulnerable to predation. The authors suggest that this difference in behaviour could contribute to the low success of this reintroduction programme. The study demonstrated the usefulness of biologging tools and how an improved understanding of species’ responses to novel environments can help to refine conservation actions.

## Looking ahead—opportunities and challenges

As outlined above, the use of biologging and biotelemetry tools has the potential to provide a mechanistic understanding of animal interactions with the environment (including human activities and infrastructure; Cooke *et al.*, 2024) and inform conservation decisions and actions. There are many opportunities that have been afforded by this ever-expanding toolbox of devices with a diverse range of sensors. As devices continue to shrink in size, one of the most obvious opportunities is the ability to tag new species and life stages. Similarly, longer battery life and enhanced storage or data compression protocols (for telemetered data) mean that animals can be monitored for longer periods. Early iterations of many biologging devices were, and still are, referred to as ‘daily diaries’ (Wilson *et al.*, 2008a) in that they detailed the lives of animals. We are now able to track animals and collect sensor data across seasons and even years, albeit this technology is still lacking compared to the use it has for humans (e.g. combination of sensors for real-time analytics on smartwatches). The longer duration of data collection is important because early studies (or even more recent ones) that used short-term deployments may suffer from effects around the capture and tag placement that can confound the data and may provide misleading information about animal behaviour or physiology. The array of sensors that is available for wildlife has increased over time (Whitford and Klimley, 2019), and we are poised to be able to measure more about the internal milieu of animals (e.g. measuring blood chemistry via nano-sensors; Lee *et al.*, 2018), sleep electrophysiology (Kendall-Bar *et al.*, 2022), as well as other biomarkers (Macdonald *et al.*, 2021). A major barrier to the use of this technology is the cost to create niche products and the limited number of companies that are willing to custom-make or modify devices for use on wildlife. Repurposing devices made for human applications is one approach for overcoming that challenge (e.g. the Medtronic devices used in the Ditmer *et al.*, 2015 and 2017 studies).

The real opportunity comes from sharing this potential with decision-makers and conservation practitioners. There are questions that we can answer today that were deemed ‘unanswerable’ just a few years ago. Fortunately, there is a growing number of studies where these tools have delivered on their promise. Indeed, we organized this paper very purposefully off of the IUCN threats and conservation interventions to make it easy for practitioners to understand what these tools have to offer.

Given the need to implement conservation actions that work, biologging and biotelemetry tools offer the potential to understand the mechanisms that drive observed patterns in population-level outcomes, like abundance, or vital statistics, like mortality (Cooke *et al.*, 2023). Another key benefit of these electronic tagging and tracking tools is the potential to generate data rather quickly, which is important when there is a need to both understand threats and evaluate conservation actions. In some cases, trends in measures like species abundance can respond relatively slowly and thus take longer to be detected and acted upon. Biologging and biotelemetry can, on the other hand, provide insight into mechanisms that drive population-level changes, allowing for more rapid decision-making. Indeed, that is regarded as one of the key benefits of the entire conservation physiology toolbox (Madliger *et al.*, 2018), of which these tagging and tracking tools are key components. Nonetheless, major challenges remain to quantify fitness impacts of threats and conservation interventions. Indeed, biologging and biotelemetry are perhaps most powerful when combined with other methods such as respirometry and tissue biochemistry (including omics). The insights that can be gained through complementary measurements are beyond the scope of this paper, but we acknowledge that there are a growing number of studies that use such approaches.

There are challenges that need to be overcome. As noted above, ethical concerns with tag placement need to be more fully acknowledged and addressed if the social licence to tag wildlife is to continue and if the data generated are to be relevant. There is also a need to document more ‘success’ stories to address the criticism that electronic tagging and tracking studies often fail to address conservation problems (McGowan *et al.*, 2017). Other challenges are more related to the ‘back end’ and involve how to deal with millions of data points (often terabytes) and the usual case where replicates (usually individuals) are low. Fortunately, there are a growing number of analytical packages and tools that streamline the processing of massive datasets (e.g. those that arise from multisensor packages that record at hundreds of Hertz). Artificial intelligence holds much promise to eventually automate more of the analysis to enable more real-time decision-making. Management decisions that are based on the tagging of just a handful of animals are often unpalatable to decision-makers and can lead them to simply ignore such studies. Therefore, the need for responsible and strategic ‘management scale’ tagging is becoming more important, which means more tags (Brownscombe *et al.*, 2019a), more animals, more data, more time and more money.

There is also a need to embrace more experimental approaches to develop cause-and-effect relationships. Many of the examples that we share in this paper are correlational or take advantage of gradients (e.g. in disturbance) that occur in the field. That certainly brings realism, but rarely captures the full picture. The creative use of controls and well-planned (or even opportunistic) experiments would certainly help to solidify biologging as a truly useful tool. Longitudinal studies across time periods of relevance to wild animals (e.g. a recent study of the longest cardiac monitoring of a free-living wild mammal—at 6 years; Laske *et al.*, 2017) and their management also hold much promise. However, biologging will always have some limitations. The study of animals in the wild and attempts to understand the additive consequences of a given anthropogenic challenge on top of the inherent challenges of life in the wild remain significant challenges. In the laboratory, it is often possible to generate rather stable and consistent physiological baselines, whereas in the wild those baselines are highly variable (laboratory behavioural controls are also a challenge to achieve given the constraints of captivity). The challenge is to determine what values to use for controls when impact and recovery are assessed, which is likely to become an increasingly important issue in the applied use of biotelemetry and biologging devices for conservation. Failure to better understand and overcome those limitations will mean that biotelemetry and biologging are viewed as ‘cool’ technologies to generate science ‘bits’ that do not translate into conservation decisions. In terms of its role to help us understand and address conservation problems, we are much further ahead than we were when a previous review was penned over a decade ago (i.e. Wilson *et al.*, 2015) as biologging and biotelemetry have become trusted tools in the toolbox of conservation physiology (Madliger *et al.*, 2018). Nonetheless, we submit that the so-called ‘golden age of biologging’ (and biotelemetry; Wilmers *et al.*, 2015) has yet to be fully realized—so stay tuned.

## Data Availability

This is a perspective article so no data were used in generating this manuscript.
